# Supplementary material for: Habitual exercise evokes fast and persistent adaptation during split-belt walking
Source: PLoS One. 2023 Jun 2;18(6):e0286649. doi: 10.1371/journal.pone.0286649 (PMC10237419; doi:10.1371/journal.pone.0286649)
Supplement: S1 Table — Note: All models contain a random plateau on participant. Model 1 contains fixed effects for the parameters in the one-exponent equation (Eq 1), fitted to the whole sample. Model 2 contains fixed effects for the parameters in the one-exponent equation, fitted by group. Model 3 contains fixed effects for the parameters in the two-exponent equation (Eq 2), fitted to the whole sample. Model 4 contains fixed effects for the parameters in the two-exponent equation, fitted by group. AIC = Akaike Information Criterion; BIC = Bayesian Information Criterion. The final model for each outcome measure is in bold (model 4 for all outcome measures). (PDF) [file pone.0286649.s002.pdf]

Table S1. Fits of mixed effects linear models for each outcome measure.

Variable	*1-exponent model*	*1-exponent + group model*	*2- exponent model*	***2-exponent + group model***
**Step length asymmetry**				
AIC	—	—	-105693.6	-106168.7
BIC	—	—	-105636.3	-106070.6
Log-likelihood	—	—	52853.78	53096.36
Number of observations	—	—	26336	26336
Number of participants	—	—	32	32
SD: ID (*c*)	—	—	0.038	0.038
SD: Residual (*c*)	—	—	0.032	0.032
**Positive work rate of the fast leg**				
AIC	2409.80	2230.21	2211.81	2021.38
BIC	2447.25	2290.13	2264.23	2111.25
Log-likelihood	-1199.90	-1107.11	-1098.90	-998.69
Number of observations	13216	13216	13216	13216
Number of participants	32	32	32	32
SD: ID (*c*)	0.441	0.435	0.441	0.435
SD: Residual (*c*)	0.263	0.261	0.261	0.259
**Negative work rate of the fast leg**				
AIC	-12275.95	-12209.38	-12285.29	-12350.91
BIC	-12238.51	-12149.46	-12232.87	-12261.04
Log-likelihood	6142.98	6112.69	6149.65	6187.46
Number of observations	13216	13216	13216	13216
Number of participants	32	32	32	32
SD: ID (*c*)	0.352	0.347	0.352	0.347
SD: Residual (*c*)	0.151	0.151	0.151	0.150
**Positive work rate of the slow leg**				
AIC	-12371.19	-12699.95	-12624.73	-12838.49
BIC	-12333.74	-12640.04	-12572.31	-12748.6
Log-likelihood	6190.59	6357.98	6319.37	6431.24
Number of observations	13216	13216	13216	13216
Number of participants	32	32	32	32
SD: ID (*c*)	0.232	0.231	0.232	0.231
SD: Residual (*c*)	0.150	0.148	0.149	0.147
**Negative work rate of the slow leg**				
AIC	5319.77	5203.29	4962.98	4848.64
BIC	5357.22	5263.21	5015.40	4938.51
Log-likelihood	-2654.89	-2593.65	-2474.49	-2412.32
Number of observations	13216	13216	13216	13216
Number of participants	32	32	32	32
SD: ID (*c*)	0.579	0.572	0.579	0.572
SD: Residual (*c*)	0.293	0.292	0.289	0.288
