# Supplementary figures and images for: Habitual exercise evokes fast and persistent adaptation during split-belt walking
Source: PLoS One. 2023 Jun 2;18(6):e0286649. doi: 10.1371/journal.pone.0286649 (PMC10237419; doi:10.1371/journal.pone.0286649)

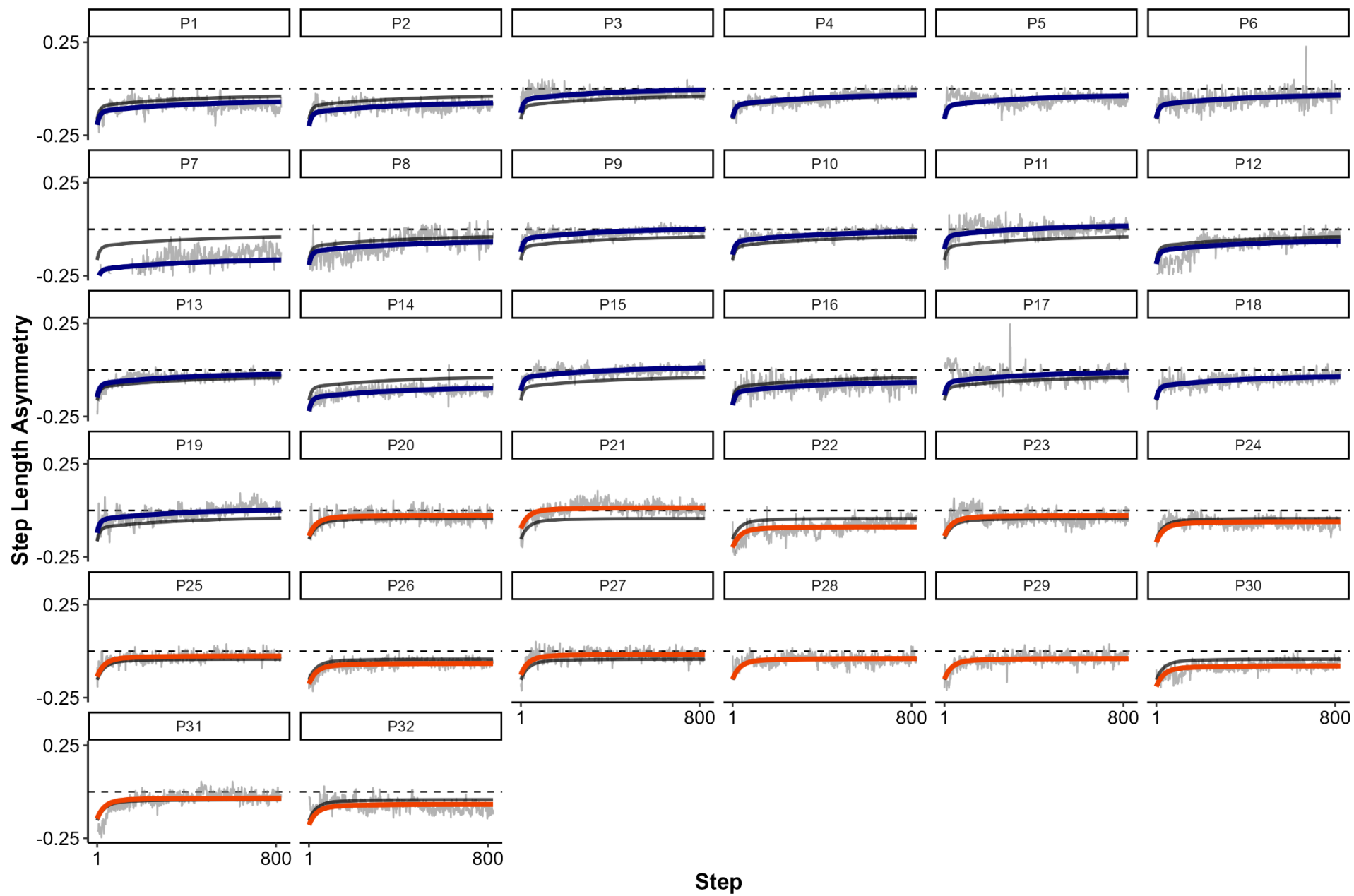

Supplement: S1 Fig — (PDF) [file pone.0286649.s003.pdf]

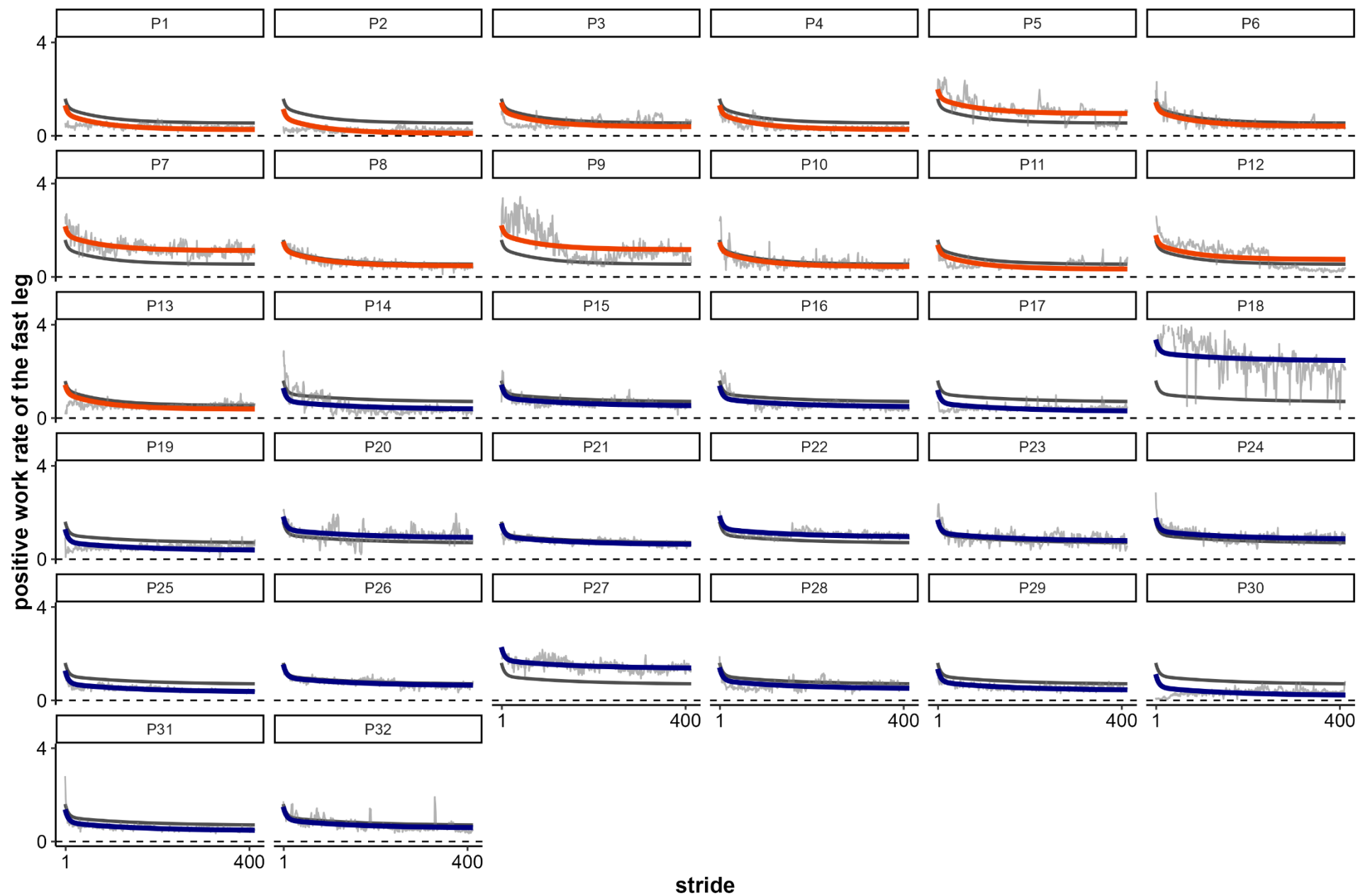

Supplement: S2 Fig — (PDF) [file pone.0286649.s004.pdf]

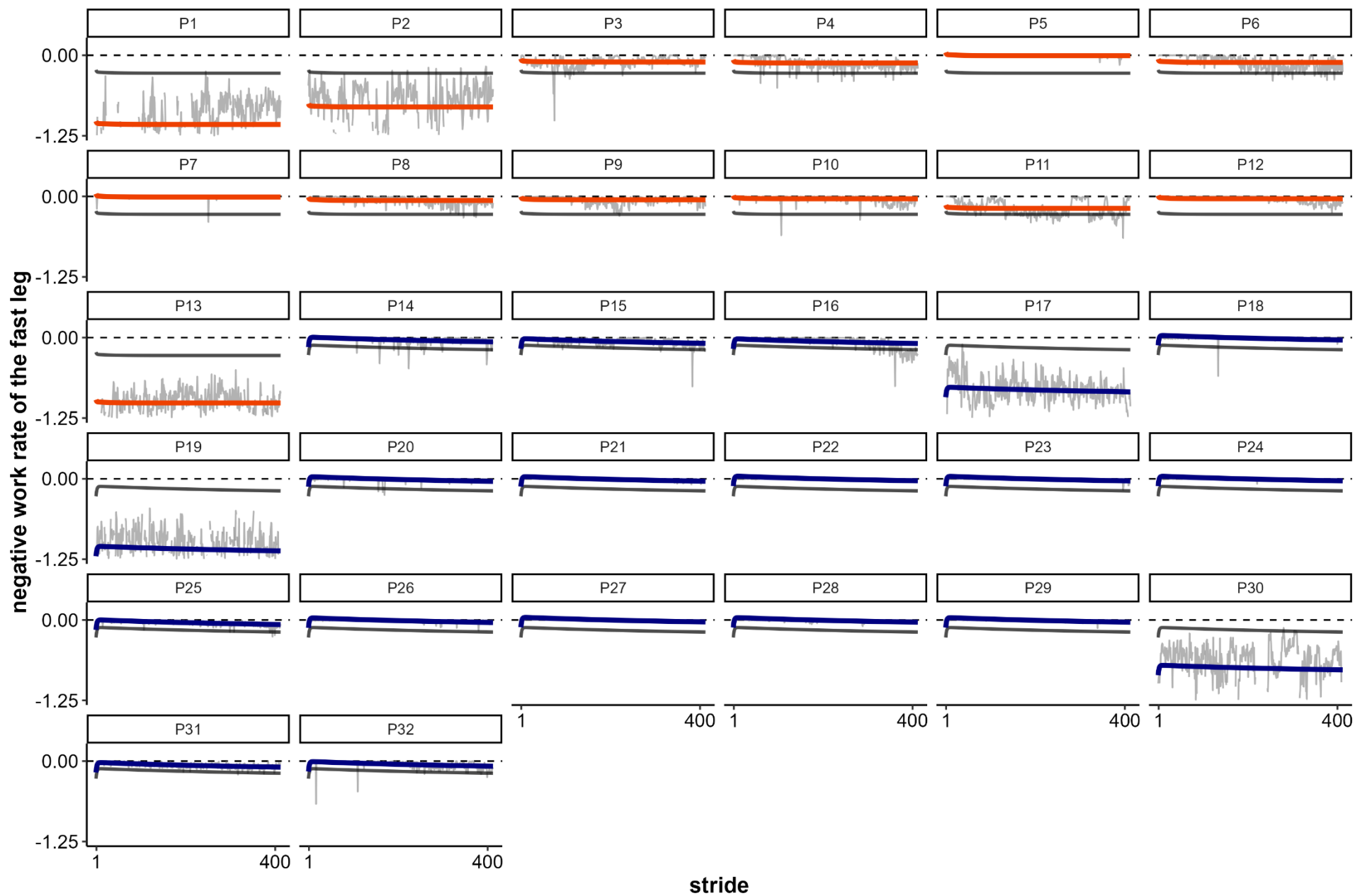

Supplement: S3 Fig — (PDF) [file pone.0286649.s005.pdf]

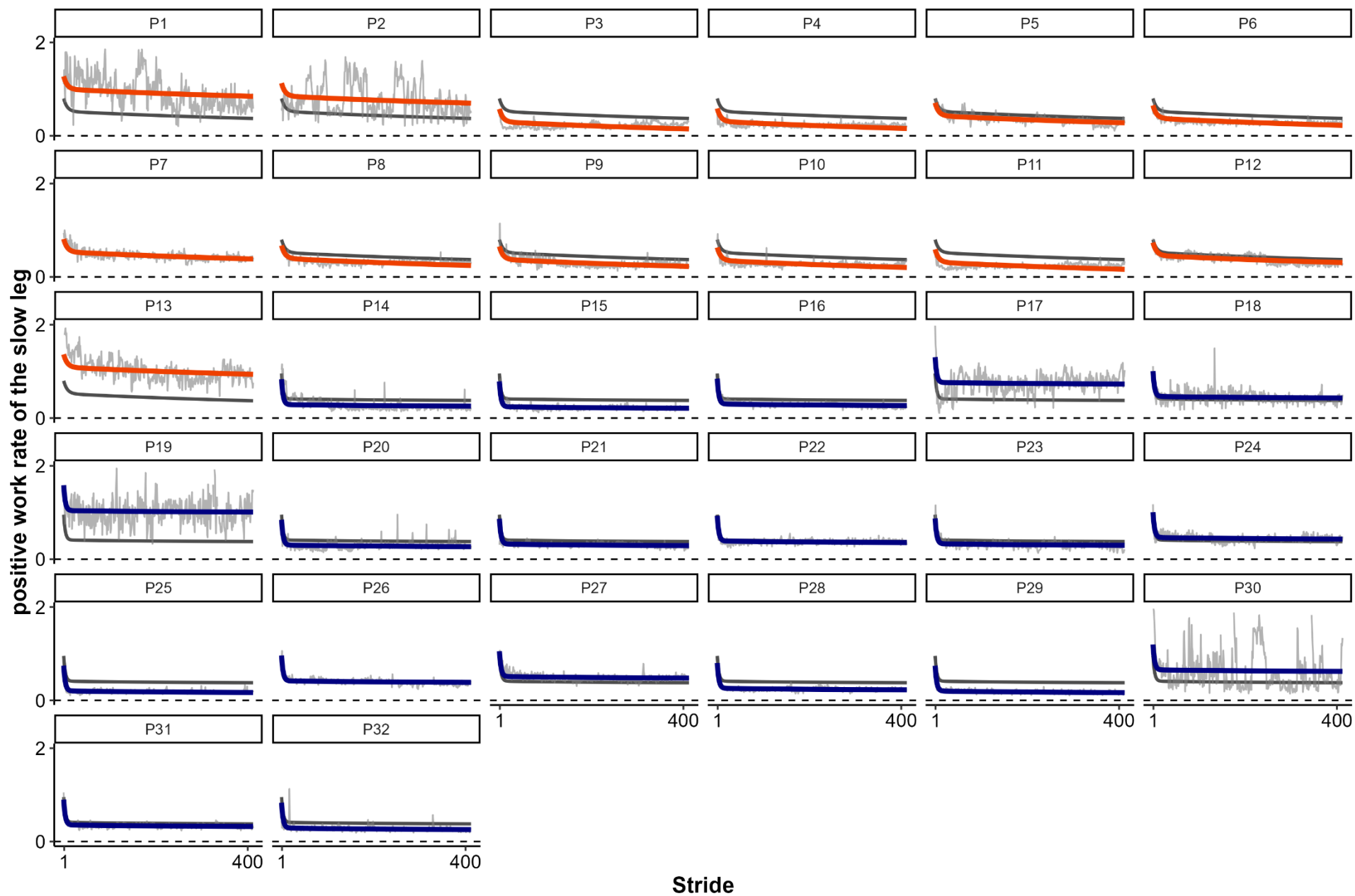

Supplement: S4 Fig — (PDF) [file pone.0286649.s006.pdf]

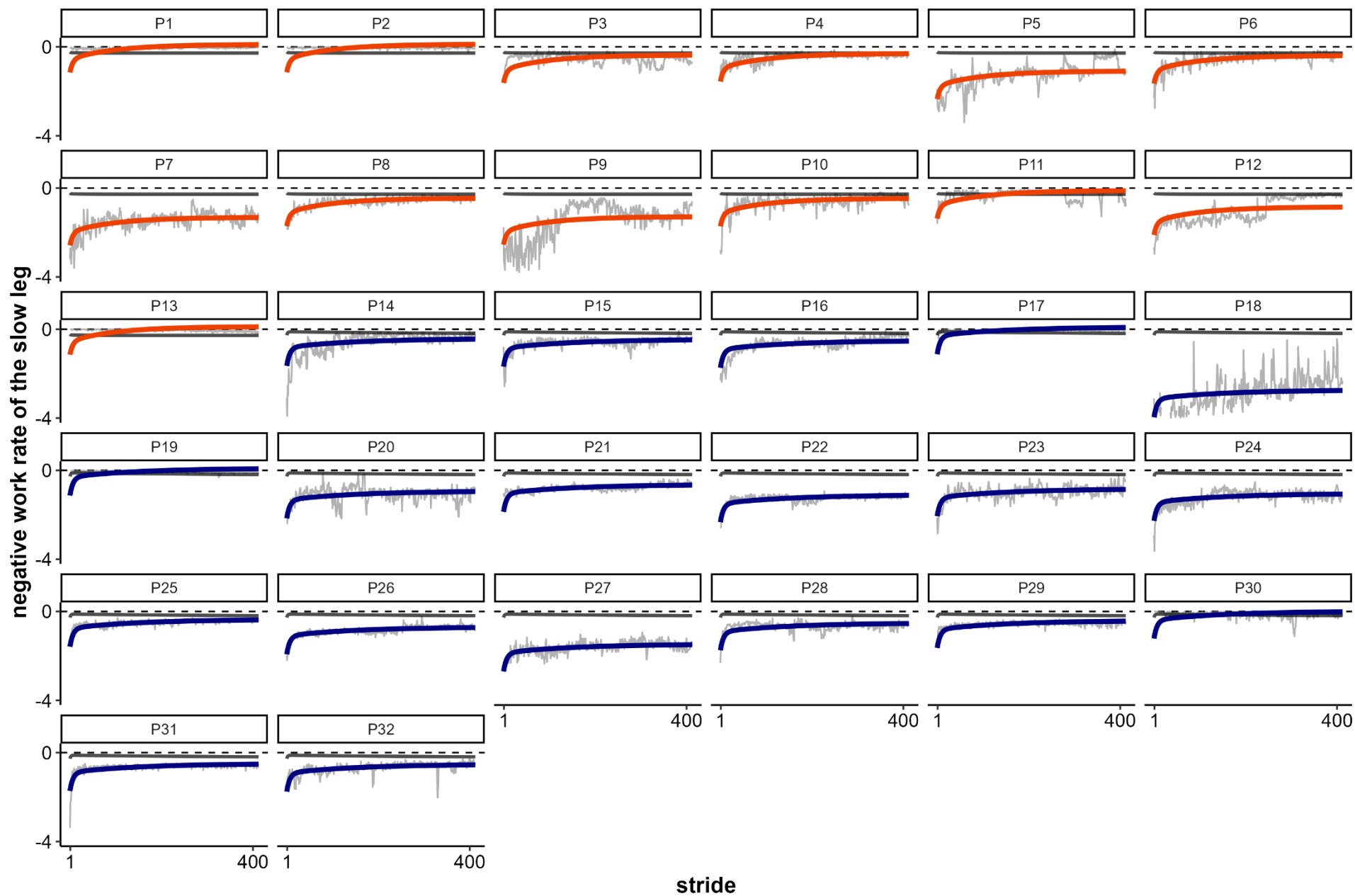

Supplement: S5 Fig — (PDF) [file pone.0286649.s007.pdf]
