# Supplementary material for: Habitual exercise evokes fast and persistent adaptation during split-belt walking
Source: PLoS One. 2023 Jun 2;18(6):e0286649. doi: 10.1371/journal.pone.0286649 (PMC10237419; doi:10.1371/journal.pone.0286649)
Supplement: Attachment — Submitted filename: Response to Reviewers_r2_v1.docx [file pone.0286649.s009.docx]

**Reviewer 1:**

This revised manuscript is much improved and in my opinion very close to being ready for publication. I only have one remaining minor point and congratulate the authors.

**Author response:**

We thank the reviewers for their careful and insightful reviews that contributed to the improvement of the manuscript.

1. ll. 354-356: Points 1 and 2 of the results summary seem a bit at odds when it is not clearly stated that 1 (if I read it correctly) refers to the slow component. It would be useful to add this information.

**Author response:**

We clarified the main findings in the discussion summary as follows:

Line 355-358: “Our main findings are: 1) Compared to notMOVE, MOVE more gradually adapted the slow timescale of SLA, positive work rate by the fast leg, and negative work rate by the slow leg over the entirety of split-belt exposure, 2) MOVE initially adapted the fast timescale of SLA and positive work rate by the slow leg quicker than notMOVE…”

**Reviewer 2:**

General: The authors showed great care in reviewing the manuscript, presenting relevant resolutions for the comments referring to the first review. The document is already in conditions to be published, with the need for small and simple adjustments.

**Author response:**

We thank the reviewers for their careful and insightful reviews that contributed to the improvement of the manuscript.

Introduction: Page 4, Line 88-90: I believe that the sentence "such that people with more aerobic training reach a minimum cost of transport while running, but those who engage in less aerobic training do not" could be improved.

**Author response:**

We have split this sentence into two and revised it for clarity.

Line 87-90: “Moreover, the ability to achieve minimum energetic cost of transport while running is contingent upon a person’s level of aerobic training experience. People who engage in more aerobic training are able to reach this optimal cost of transport, whereas people with less aerobic training are not.”

Introduction: Page 4, Line 93-96: I'm not sure the word "conversely" (Line 95) is used correctly. After all, it seems to me that the two statements that the word connects are not in disagreement. In fact I understood that the second sentence only justifies the first, in which individuals who engage in more exercise achieve an optimal energy cost due to greater submaximal tolerance.

**Author response:**

The difference between the two statements is in the rate of adaptation due to exercise training – the first suggests that exercise-trained people adapted quicker, the second suggests that exercise-trained people adapt slower. These two hypotheses propose competing results, and we test which is more likely to be true in this study. We have revised this statement to hopefully read more clearly.

Line 93-98: “In this study, we examine two competing hypotheses regarding the adaptation to split-belt walking and its impact on energetic cost. The first hypothesis suggests that individuals who engage in more exercise would reach an energetic optimum faster, aiming to reduce energetic cost [28]. Conversely, the second hypothesis suggests that individuals who engage in more exercise may more gradually approach an energetic optimum due to their greater tolerance for submaximal exercise [31,32].”

Discussion: Page 19, Line 425-432: I believe this passage is out of place. In my view part of it should be in the methods section and the other part in the results section

**Author response:**

In response to the reviewer's comment regarding the placement of a particular section in our manuscript, we respectfully disagree with their suggestion. The mentioned section does not pertain to a specific question or hypothesis of the paper, but rather serves as a follow-up analysis aimed at determining the validity of a discussion point. As such, we believe it is more appropriately placed within the discussion section, where we critically analyze and interpret our findings in relation to the broader context of the study. We believe that including this analysis in the methods and results sections would disrupt the flow and coherence of our manuscript.
