# Supplementary material for: Habitual exercise evokes fast and persistent adaptation during split-belt walking
Source: PLoS One. 2023 Jun 2;18(6):e0286649. doi: 10.1371/journal.pone.0286649 (PMC10237419; doi:10.1371/journal.pone.0286649)
Supplement: Attachment — Submitted filename: Response to Reviewers_r3_v1.docx [file pone.0286649.s010.docx]

**Additional Editor Comments:**

Dear Authors,

I congratulate you by your study. Before a final acceptance, please consider describing the add-hoc analysis in results section, then you may briefly discuss these results in the discussion section, making the discussion a bit shorter. Thank you!

**Author Response:**

Thank you for your prompt review of our manuscript. We revised the post-hoc analysis so that the bulk of the information is in the results section (lines 307-314) and shortened the discussion section containing these results (lines 430-442).
